# Evolution of hemostatic agents in surgical practice

**DOI:** 10.4103/0970-1591.70574

**Published:** 2010

**Authors:** Chandru P. Sundaram, Alison C. Keenan

**Affiliations:** Department of Urology, Indiana University School of Medicine, Indianapolis

**Keywords:** Fibrin sealant, gelfoam, hemostatic agents, microfibrillar collagen, oxidized regenerated cellulose

## Abstract

**Objective:**

Topical hemostatic agents are used in a wide variety of surgical settings, and the evolution of this class of surgical tools is an interesting topic. We reviewed and outlined the historical progress of topical hemostats into present day surgery and urology, and highlight opportunities for future research.

**Materials and Methods:**

A MEDLINE search of all available literature concerning several classes of topical hemostatic agents was performed. Fibrins sealants, Gelatin sponge hemostatics, cyanoacrylate adhesives, oxidized regenerated cellulose, and microfibrillar collagen were included. References were chosen from a broad range of surgical literature.

**Results:**

Topical hemostatic agents have historically taken advantage of a wide variety of mechanisms for hemostasis. Fibrin sealants have a rich history and large potential for further applications. Gelatin sponge hemostatics have been widely used since their introduction, but have changed little. Cyanoacrylate adhesives have a unique mechanism and opportunity for novel applications of existing products. Oxidized cellulose was original in the use of plant-based components. Microfibrillar collagen hemostats have evolved to a wide variety of formats.

**Conclusions:**

A review of the evolution of topical hemostatic agents highlights opportunities for potential novel research. Fibrin sealants may have the most opportunity for advancement, and understanding the history of these products is useful. With the drive in urology for minimally invasive surgical techniques, adaptation of topical hemostatic agents to this surgical approach would be valuable and offers an opportunity for novel contributions.

## INTRODUCTION

As any surgeon would agree, hemostasis is of critical importance during all surgical procedures. A fundamental principle of good surgical technique is minimization of blood loss, and present day surgeons have a wide variety of agents and tools to aid them in this endeavor. Few urologic surgeons would be eager to undertake renal procedures without the ubiquitous electrosurgical unit. Although used less frequently than simple electrocautery, topical hemostatic agents are useful in minimizing blood loss and in turn surgical morbidity. Although no aid in hemostasis can negate the importance of good surgical technique, even the most talented surgeon has encountered persistent bleeding which has required focused attention. Often a topical hemostatic agent is helpful in these situations, and the evolution of these agents is an interesting topic in surgical history. In this article, we review the history and evolution of selected hemostatic agents and highlight areas with potential for future advancements. Special attention is paid to the fibrin sealants, which have an interesting history and significant potential for novel urologic research.

## A REVIEW OF HEMOSTASIS

Hemostasis is a complex process requiring the delicately coordinated activation of platelets and plasma clotting factors to form a platelet-fibrin clot.[1] This can be divided into two distinct processes, primary and secondary hemostasis. Primary hemostasis results in the formation of soft platelet plugs, which in turn are stabilized and cross-linked during secondary hemostasis. Of central importance in both primary and secondary hemostasis is the activation of the clotting cascade, which can be broken down into two basic pathways, the intrinsic pathway and the extrinsic pathway.[2] The intrinsic pathway is activated by collagen, which is exposed when a blood vessel is damaged. The extrinsic pathway is similarly activated by tissue damage and the resultant release of tissue factor. The intrinsic and extrinsic pathways converge into the common pathway, which begins with the conversion of Factor X to Xa, and ultimately results in the conversion of prothrombin to thrombin, which is integral in clot stabilization via fibrin.[3] The common pathway is facilitated by Factor V, which surgeons often think of due to the relatively common heritable coagulation disorder, Factor V Leiden.[4] The physiology of hemostasis is well-understood, and historically many topical hemostatic agents have been designed to mimic or exploit the enzymes and molecules central to the process.

## FIBRIN SEALANTS

Topical hemostatic agents in the modern surgical era can be traced to 1909, when Bergel first discussed the use of topical fibrin for hemostasis.[5] This evolved into a class of preparations known as fibrin sealants. Early fibrin sealants combined bovine thrombin with human plasma for topical application.[6] Widespread surgical usage was initially limited, but in 1938 purified thrombin became available through advancements in the technology of protein separation.[7] That technology accelerated research and development of fibrin sealants, and reports of apparently successful usage of such agents began to appear in the surgical literature in the 1940s. Young and Medawar reported the use of fibrin sealant to repair peripheral nerves in 1940.[8] In 1944, Cronkite *et al*. reported the usage of combined fibrinogen and thrombin in improving skin graft survival and adhesion in the grafting of soldiers with severe burn injuries.[9]

Although exciting, the early formulations were fraught with problems, namely their potentially infectious nature. All early fibrin sealants were derived from human plasma components, and as with all human-derived products at the time, preparations of human fibrinogen and thrombin were a source of transmission of viral hepatitis, and some early patients treated with fibrin-based hemostatic agents contracted hepatitis. In an effort to minimize the risk of viral transmission, bovine thrombin was substituted for human thrombin in many subsequent preparations. This resulted in some patients developing coagulopathies associated with the use of bovine thrombin related to immune-mediated production of thrombin and Factor V inhibitors.[10,11] The risk of viral transmission, in addition to the coagulopathies, seen with bovine thrombin usage led to significantly decreased appeal, and work on these sealants essentially ceased for a number of years.

Interest was revived in the late 1960s with advancements in technology allowing for the isolation and concentration of clotting factors from human plasma. Cryoprecipitate became widely available, and in 1972, Matras combined cryoprecipitate with purified bovine thrombin to produce the first modern iteration of a fibrin sealant.[12] Research continued, and the first commercially available fibrin sealant was approved in Europe in 1982. Despite reported success among European and Japanese surgeons with use of fibrin sealants, the American Food and Drug Administration (FDA) did not follow in approving the product because of the perceived risk of viral transmission from the pooled plasma component.[7] As use by foreign surgeons continued, American surgeons implemented the use of noncommercial forms of fibrin sealant throughout the 1990s. Most of these surgeons generated sealants which were created through combining patient’s own plasma clotting factors through autologous donation, with bovine thrombin.[13] Progress was made in reducing the risk of viral transmission through human blood products, and ultimately in 1998, Tisseel and Hemaseel fibrin sealants were approved for clinical use by the American FDA. The most recent variation on fibrin sealants came in 2003, when the American Red Cross, in conjunction with OMRIX Biopharmaceuticals, released Crosseal, a fibrin sealant manufactured entirely from nonanimal components. Crosseal contains human fibrinogen and thrombin, and lacks the bovine aprotinin component found in the other fibrin sealants.[14]

Published reports of clinical applications for fibrin sealants were initially largely limited to cardiac and vascular surgery, oral and maxillofacial surgery, and reconstructive plastic surgery, but recently, more and more applications are being discussed in the urologic literature. Mueller *et al*. presented an interesting series of eight patients in which the fibrin sealant Tisseel was used to create ureteral obstruction during laparoscopic pluck nephroureterectomy.[15] As a hemostatic agent, Zhang *et al*. published a modified technique of renal artery anastamosis utilizing fibrin sealant in a series of renal transplantations in rats.[16] Gopal *et al*. and Ambriz-Gonzalez *et al*. reported decreased fistula and complication rates, respectively, when fibrin sealants are used over suture lines during hypospadias repair.[17,18] Further publications in the urologic literature report the utility of fibrin glue in tubeless percutaneous renal surgery, as well as in the repair of complicated vesicovaginal fistulas as an alternative to the martius flap.[19,20]

Although fibrin sealant was one of the first modern hemostatic agents to be used in clinical practice, the slow development of the technology and initial obstacles to FDA approval and subsequent limited availability have lead to a relative paucity of prospective clinical research with fibrin sealants. The utility of effective hemostasis in a topical biologic agent combined with effective tissue sealant is of undeniable interest to any urologic surgeon. As an understudied material, fibrin sealants offer an exciting arena of potentially novel clinical urologic research. Given that Tisseel and other sealants are now widely commercially available, the potential for large prospective randomized clinical trials in common urologic procedures, such as percutaneous renal surgery and ureteral anastamosis, is quite exciting. Perhaps even more interesting than the hemostatic uses of fibrin sealants are the therapeutic possibilities. The potential for further evolution in the application of fibrin sealants has been shown in biomaterials research utilizing fibrin sealants for topical delivery of antibiotics, chemotherapeutics, and analgesics, which offers yet another interesting opportunity for novel research.[21]

## GELATIN HEMOSTATIC AGENTS

Fibrin sealants were one of the first modern hemostatic agents, but their initially limited use opened the door for other novel hemostatic products, many of which are now more familiar to most surgeons. Gelatin-based hemostatic agents are abundant in modern operating rooms, and were first introduced in the 1940s as Gelfoam. In their fundamental form, gelatin-based hemostatic agents have undergone very little evolution since their introduction. Gelfoam and Surgifoam are still offered in similar preparations as their initial release. Gelfoam is a purified pork skin gelatin, and its hemostatic properties, although not entirely understood, are felt to be more physical rather than related to direct effects on the clotting cascade.[22] Gelfoam can be used in several ways, either in dry sponge form, moistened with injectable sodium chloride solution, or, commonly, saturated with topical purified thrombin. The combined application with topical thrombin is one of the few advancements made with gelatin-based hemostatic agents. The addition of thrombin can enhance the hemostatic properties of gelatin sponge hemostatics, but plain Gelfoam or Surgifoam is affordable and readily accessible. It is considered a mediocre hemostat by some surgeons, but has been noted to have success when used in its plain form for hemostasis in tubeless percutaneous nephrolithotomy.[23]

A property of gelatin sponge hemostatics is the ability to absorb upwards of 40 times its weight in blood or fluids, and its capacity to expand up to 200% *in vivo*.[22] This can be construed as a negative property in some settings. Another major advancement in the field of gelatin-based hemostatic agents came in the development of the product Floseal. Approved for commercial use in the US in 1999, Floseal combines human-derived thrombin with bovine-derived gelatin matrix granules which are mixed at the time of use.[24] The major evolution of this product is the liquid nature of Floseal vs gelatin sponge, as well as a novel crosslinking of the gelatin matrix granules that minimizes *in vivo* expansion of the product.[25] Although this is not an important property of a hemostatic agent in some surgical approaches, the liquid nature of the product does facilitate application, especially in minimally invasive surgery. As with gelatin sponge agents, Floseal is absorbable and well-tolerated. Rare reports of abscess formation or granuloma formation have been reported with the use of gelatin-based agents; however, some of these have been attributed to the radiologic appearance on postoperative imaging of gelatin sponges.[26,27] Accessibility, ease of use, and effective hemostasis make gelatin-based hemostatic agents a popular tool in reducing surgical morbidity from blood loss.

## CYANOACRYLATE ADHESIVES

Cyanoacrylate adhesives were developed by Dr. Harry Coover of Kodak Laboratories in 1942, during experiments to design a clear plastic suitable for gun sights. The material was unsuccessful in this application, and was abandoned.[28] In 1949, Airdis synthesized cyanoacrylate, and recognizing its unique properties, submitted the material for US patent.[29] In 1959, Coover *et al*. reported on the adhesive properties of cyanoacrylates, and suggested their application as surgical adhesives.[30] In 1965, Watson and Maguda used cyanoacrylate adhesive for tympanic membrane repair, and there were reports that cyanoacrylates were used as tissue adhesives and hemostatic agents on battlefields during the Vietnam War.[31] Eastman submitted an application for approval to the FDA for cyanoacrylates as a wound adhesive in 1964, but issues with tissue irritation and brittleness prevented approval. Finally, in 1998, after significant redesign, cyanoacrylates were approved by the FDA in the form of 2-octyl cyanoacrylate. Marketed as Dermabond, 2-octyl cyanoacrylate, has been shown to be a good tissue adhesive, and to have antimicrobial properties.

Liquid cyanoacrylate consists of cyanoacrylate monomers, which polymerize into long chains in the presence of hydroxyl ions. Water containing human tissue activates the polymerization of cyanoacrylate monomers, and is bonded together as the glue rapidly sets. The property of nearly instantaneous bonding makes cyanoacrylates an effective hemostatic agent and tissue adhesive. Currently, multiple brands and formats of cyanoacrylate adhesives are available, and are widely used for corneal perforation repair, skin laceration repair, esophageal and gastric variceal repair, skin grafting, and vascular repair. Testini *et al*. reported significantly reduced morbidity when using human fibrin glue or N-butyl-2-cyanoacrylate adhesive for mesh fixation during inguinal hernia repair when compared with suture.[32] Elmore *et al*. report the use of 2-octyl-cyanoacrylate in sutureless circumcision with good result, negating the risk of suture tracks or sinuses.[33] Aning *et al*. recently reported a novel use of cyanoacrylate adhesive for the percutaneous ablation of a late urinary fistula after partial nephrectomy.[34] Cyanoacrylates are becoming increasingly popular for the sutureless closure of small skin incisions, and as surgeons from all fields become more comfortable with the properties of these adhesives, cyanoacrylates are likely to be put to increasingly innovative uses.

## OXIDIZED REGENERATED CELLULOSE

A new iteration of topical hemostatic was launched into the clinical market in 1960 with the release of Surgicel. This topical agent was entirely unique in the market, as it was not derived from human or animal sources. Surgicel is a plant-based topical hemostatic, made by regenerating pure plant-derived cellulose into a knitted fabric which is then oxidized.[35] The oxidized cellulose fabric can be topically applied and acts as a scaffold for clot formation. Oxidized regenerated cellulose topical hemostatic agents have, like gelatin-based products, evolved little since their introduction, but are still quite effective and commonly used. Surgicel bolsters are a popular adjuvant to controlling surgical bleeding during partial nephrectomy.[36] Another common feature between the oxidized cellulose hemostatics and gelatin-based hemostatics is an appearance on postoperative imaging that can be concerning for abscess.[37] Surgicel can in fact have a ring enhancing appearance on computed tomography scan, and perhaps some of the most clinically significant advancements in the use of this hemostatic are in better understanding of its radiological features, thereby reducing the morbidity of unnecessarily intervening on a presumed abscess.

## MICROFIBRILLAR COLLAGEN

A more recently developed and approved addition to the market of hemostatic agents is collagen-based products. Avitene, a topical microfibrillar collagen hemostatic, was introduced in the US in the early 1970s. Microfibrillar collagen products are made by purifying bovine collagen and processing it into microcrystals, which can then be manipulated into hemostatic agents in a wide variety of formats. The brand Avitene was first launched in a flour form, and it is still commonly used in the topical powder form. All manner of collagen-based hemostatics enjoyed widespread early use as they were felt to be more effective than gelatin-based hemostats. Collagen-based products activate the intrinsic pathway of the coagulation cascade, whereas gelatin hemostats are thought to induce hemostasis through physical properties alone.[38] In several randomized trials, primarily in the cardiothoracic surgical literature, microfibrillar collagen hemostats have been shown to be superior to oxidized cellulose in hemostasis, and indeed were shown to have statistically significant reductions in measured blood loss when compared with oxidized cellulose.[38] The primary improvements and changes made in the field of collagen hemostatics have been in the release of new formats. As previously stated, the first collagen hemostat, Avitene was initially available in a flour form, and today the product line has expanded to a nonwoven web material, as well as a sponge format. In May 2000, the most recent addition, the sponge form Avitene Ultrafoam Collagen, was approved by the American FDA.[39]

## SUMMARY

Fibrin sealants, gelatin-based products, oxidized cellulose, and collagen products are the major classes of topical hemostatic agents that have evolved over the last 100 years. Three of these classes have been relatively static since their development, with changes mostly to format and preparation. The evolution of fibrin sealants, conversely, has been slow but steady over the last century, and holds the great promise for future adaptations. This is especially exciting, when considered in the context of current surgical practice. Surgery of all kinds, but even more so Urologic surgery, is undergoing a period of rapid growth and evolution of techniques, instrumentation, and theory, driven by the modern push for minimally invasive surgery. As new devices and surgical systems reach clinical practice with impressive speed, there needs to be a concurrent pressure for the adaptation of surgical tools and drugs. Hemostasis is always critically important in any surgical approach, and it can prove particularly challenging in minimally invasive surgery. For example, in laparoscopic partial nephrectomy, control of surgical bleeding can be the most technically challenging step in the operation.[40] Well-designed, effective topical hemostatic agents are invaluable in minimally invasive surgical practice. Multiple different mediums and classes of topical hemostatics are prime subjects for adaptation to urologic surgical approaches of the future. In particular, fibrin sealant hemostatic agents have a rich history and large potential for further reinvention.
